# Activated Interferon‐γ‐Positive T Lymphocytes and Cytokine Signatures in Patients With Postinfectious Cough

**DOI:** 10.1002/mco2.70340

**Published:** 2025-08-24

**Authors:** Zheng Deng, Tongtong Song, Wenbin Ding, Wei Luo, Jiaxing Xie, Haodong Wu, Nanshan Zhong, Kefang Lai

**Affiliations:** ^1^ State Key Laboratory of Respiratory Disease Guangzhou Institute of Respiratory Disease The First Affiliated Hospital of Guangzhou Medical University Guangzhou China; ^2^ Guangzhou National Laboratory Guangzhou China

**Keywords:** airway inflammation, cytokines, immune responses, postinfectious cough, T lymphocytes

## Abstract

Postinfectious subacute cough (PISC) and postinfectious chronic cough (PICC) are triggered by respiratory infections, which induce adaptive immunity. The expression of T‐lymphocyte subsets and cytokine signatures remains elusive in these patients. Here, we recruited 40 healthy controls, 64 PICC patients, 65 PISC patients, and 20 recovered individuals with postinfectious subacute cough (R‐PISC). As cough and airway inflammation resolved in R‐PISC subjects, sputum lymphocytes dropped substantially. Both PICC and PISC patients had an increase in blood activated interferon‐γ (IFN‐γ)^+^ T‐lymphocytes, which were decreased in R‐PISC subjects. Elevated cough sensitivity, higher proportions of activated IFN‐γ^+^ T‐lymphocytes, and CD8^+^/CD4^+^ T‐lymphocyte ratios, as well as elevated concentrations of uric acid, IFN‐γ, tumor necrosis factor‐α (TNF‐α), IFN‐α, IFN‐β, and interleukin‐10 in sputa, were observed in PICC and PISC patients but normalized in R‐PISC subjects. Correlation analyses and logistic regression models identified activated IFN‐γ^+^ T‐lymphocytes and these cytokines in sputa as biomarkers for predicting cough risk. PICC patients exhibited greater cough severity, elevated activated IFN‐γ^+^ T‐lymphocytes, and TNF‐α concentrations in sputa compared to PISC patients. Overall, postinfectious cough patients exhibit airway inflammatory signatures characterized by activated IFN‐γ^+^ T‐lymphocytes and elevated levels of IFN‐γ, TNF‐α, IFN‐α, IFN‐β, and interleukin‐10, which are valuable for effective treatment options.

## Graphical abstract

Postinfectious cough patients have elevated levels of activated IFN‐γ^+^ T lymphocytes in blood. Postinfectious cough patients exhibit airway inflammatory signatures characterized by activated IFN‐γ^+^ T‐lymphocytes and elevated levels of IFN‐γ, TNF‐α, IFN‐α, IFN‐β, and IL‐10. Postinfectious chronic patients present with a more severe cough phenotype than postinfectious subacute cough patients.

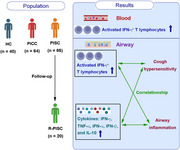

## 1 Introduction

Cough is a predominant symptom in respiratory infections. Postinfectious cough, representing the predominant etiology of subacute cough, develops in approximately 11%–25% patients following upper respiratory tract infections. Approximately 2.8% of patients with postinfectious acute cough progress to chronic cough [1]. Patients with postinfectious subacute cough (PISC) typically present with persistent cough lasting 3–8 weeks following resolution of acute respiratory infection symptoms. The cough duration extends beyond 8 weeks in patients with postinfectious chronic cough (PICC) [2]. Chest radiographs demonstrate no detectable abnormalities in either PISC or PICC patients [3]. Viral pathogens are responsible for 90% of postinfectious cough cases [4]. Among viruses causing acute respiratory infections, influenza A viruses account for the highest percentage (34.9%), followed by rhinovirus (20.5%), respiratory syncytial virus (12.8%), influenza B viruses (8.3%), and adenovirus (7.6%) [5]. About 20.9% and 4.7% of patients with a new emerging coronavirus disease 2019 infection progress to PISC and PICC patients [6]. Viral infections result in respiratory epithelial damage, innate and adaptive immune responses associated with inflammatory cell infiltrations. Exuberant T lymphocyte responses and excess cytokine productions drive dysregulated adaptive immune responses, leading to lung inflammation [7]. Viable respiratory viruses are generally cleared from patients' lungs within 1–2 weeks after symptom onset [8, 9]. Treatment options for postinfectious cough (including corticosteroids and bronchodilators) demonstrate no clinically significant benefit to these patients [10]. Central acting antitussive agents such as codeine and dextromethorphan are considered to treat postinfectious cough when other drugs fail [3]. Advancing our mechanistic understanding of postinfectious cough pathogenesis represents a critical unmet need for developing targeted therapeutic strategies.

Cluster of differentiation 3^+^ (CD3^+^) T‐lymphocytes can be divided into CD8^+^ T‐lymphocytes and CD4^+^ T helper (Th) lymphocytes. Effects of CD8^+^ T lymphocytes, such as cytotoxicity and production of the pro‐inflammatory cytokines (interferon‐γ [IFN‐γ] and tumor necrosis factor‐α [TNF‐α]), contribute to influenza‐induced lung immunopathology [11]. Based on cytokine secretion patterns, CD4^+^ Th lymphocytes polarize into Th1, Th2, Th17, and other subsets. Th1 lymphocytes secrete IFN‐γ and TNF‐α. Type I interferons (IFN‐α and IFN‐β) promote lymphocyte activation and subsequent IFN‐γ production [12]. IFN‐γ^+^ T‐lymphocyte populations and IFN‐γ production can be substantially upregulated by the infection of influenza A virus, rhinovirus, respiratory syncytial virus, influenza B virus, and adenovirus [13, 14, 15, 16, 17]. Activated CD8^+^ T lymphocytes, IFN‐γ^+^ T lymphocytes, and IFN‐γ production are persistently elevated in some patients with persistent, distressing symptoms following an infection with severe acute respiratory syndrome coronavirus‐2 [18]. IFN‐γ^+^ T lymphocytes play an important role in inducing cough hypersensitivity [19]. Th2 lymphocytes produce interleukin‐4 (IL‐4) and IL‐5. Th17 lymphocytes are characterized by their production of IL‐17 and can be differentiated into effector cells through the synergistic action of IL‐23, IL‐1β, and IL‐6 [20, 21]. A distinct subpopulation of CD4^+^ T lymphocytes can produce IL‐8 [22]. IL‐10 is secreted by all T‐cell subsets. Of note, IL‐10 can enhance the proliferation of CD8^+^ T lymphocytes and their production of IFN‐γ [23]. Animal studies suggest that cough can be induced by many cytokines via activating vagal sensory neurons, including IFN‐γ, IFN‐α, IFN‐β, IL‐1β, and TNF‐α [24, 25, 26].

Interferon‐γ‐inducible protein 10 (IP‐10) is a chemokine ligand that binds to CXC chemokine receptor 3 (CXCR3), a receptor expressed on activated T lymphocytes and other cell types [27]. The IP‐10/CXCR3 pathway mediates influenza virus‐induced expansion of activated CXCR3^+^IFN‐γ^+^ T lymphocytes and IFN‐γ production, ultimately driving pulmonary inflammation [13]. Respiratory viral infection upregulates expression of human leukocyte antigen DR (HLADR), a late‐phase activation marker of lymphocytes [28]. Regardless of the respiratory viral pathogen type, T cells are activated by antigen‐presenting cell‐presented viral antigens via T‐cell receptors [29]. Meanwhile, many T cells are activated in a viral antigen‐independent and cytokine‐dependent bystander mechanism. Virus‐induced secretion of cytokines (including IFN‐α and IFN‐β) mediate the bystander activation of HLADR^+^CD8^+^ T lymphocytes, thereby promoting immunopathology [30]. Persistent bystander T‐cell activation with the ability to secret IFN‐γ plays an important role in patients with unresolved inflammation despite the decline in viral loads [29]. CC chemokine receptor 4 (CCR4) expression by Th2 lymphocytes contributes to the pathogenesis of allergic inflammation [31]. Currently, no study has characterized T‐lymphocyte subset profiles and associated cytokine patterns in patients with postinfectious cough.

Addressing the critical need to characterize T‐lymphocyte subsets and cytokine profiles in patients with postinfectious cough, we comprehensively analyzed clinical characteristics, peripheral blood and induced sputum immunophenotypes (T‐lymphocyte subsets and cytokines), and their associations with cough sensitivity and airway inflammation across four study groups: healthy controls, PICC patients, PISC patients, and recovered individuals with postinfectious subacute cough (R‐PISC). We further developed logistic regression models to predict cough risk factors in patients with postinfectious cough.

## 2 Results

### 2.1 Characteristics of the Study Population

As shown in Table 1, this study finally recruited 40 healthy controls, 64 PICC patients, 65 PISC patients, and 20 R‐PISC subjects. No significant differences were observed in age, sex, or BMI across the four groups. PISC patients and R‐PISC subjects at the period of PISC (at baseline) showed similar demographics and clinical characteristics (Table S1), suggesting that the recruited R‐PISC subjects might represent all the enrolled PISC patients that had completely recovered in this study. VAS scores showed no significant difference between PICC patients (median score of 60.0) and PISC patients (median score of 60.0). LCQ scores were significantly lower in PICC patients (median score of 11.9) compared to PISC patients (median score of 13.0), indicating a worse cough‐specific life quality in PICC patients. The prevalence of dry cough was higher in PICC patients (62.5%) versus PISC patients (43.1%). Comparative analysis revealed no statistically significant variations in the percentages of circulating neutrophils, lymphocytes, or eosinophils between different groups.

**TABLE 1:** Demographics and clinical characteristics of subjects.

	HC	PICC	PISC	R‐PISC
Total number of subjects	40	64	65	20
Age (years)	44.0 (25.5–51.8)	37.0 (29.3–50.0)	36.0 (31.0–45.0)	34.5 (31.3–40.8)
Female	20 (50%)	38 (59.4%)	42 (64.6%)	12 (60%)
BMI (kg/m^2^)	22.0 (20.5–24.2)	23.0 (21.0–24.0)	22.0 (20.0–24.2)	22.0 (20.0–24.8)
Cough duration (weeks)	─	66 (36–156)^†††^	7 (4–8)	─
Dry cough	─	40 (62.5%)^†^	28 (43.1%)	─
**Cough symptom evaluation**				
Cough VAS	─	60.0 (50.0–70.0)	60.0 (42.5–80.0)	─
Daytime cough scores	─	3 (3–4)	3 (3–4)	─
Night‐time cough scores	─	1 (1–2)	1 (1–2)	─
LCQ scores	─	11.9 (9.9–13.5)^†^	13.0 (12.0–13.9)	─
**Blood**				
Neutrophils (%)	54.60 (51.70–62.70)	59.40 (54.10–64.33)	59.55 (54.03–66.18)	60.50 (52.25–64.40)
Eosinophils (%)	1.30 (1.00–2.30)	1.55 (0.88–3.43)	1.55 (1.03–2.85)	2.00 (1.20–2.95)
Lymphocytes (%)	33.39 ± 7.89	31.95 ± 7.08	31.38 ± 7.82	30.91 ± 7.26
**Induced sputum**				
Neutrophils (%)	53.00 (33.69–67.94)	62.25 (44.50–81.38)	62.50 (34.19–84.00)	64.88 (47.38–76.00)
Macrophages (%)	45.63 (30.73–64.95)	35.50 (16.00–54.50)	33.50 (13.75–61.63)	28.63 (18.13–46.81)
Eosinophils (%)	0 (0–0.50)	0.37 (0–0.81)	0.25 (0–1.13)	0.25 (0–0.50)
Lymphocytes (%)	1.45 (0.74–1.81)	1.25 (0.50–2.00)	1.63 (0.69–3.00)	0.50 (0–1.44)^##^
**Spirometry**				
FVC% of predicted (%)	99.9 ± 9.7	100.3 ± 15.6	102.3 ± 10.6	─
FEV1% of predicted (%)	100.0 ± 9.9	99.6 ± 15.1	100.8 ± 10.0	─
MMEF% of predicted (%)	82.0 (69.0–99.0)	79.3 (59.1–91.0)	82.7 (68.3–97.5)	─
FEF50% of predicted (%)	90.9 ± 26.1	82.7 ± 23.2	89.2 ± 20.8	─
FEF75% of predicted (%)	81.0 (62.5–99.5)	69.2 (50.2–87.5)^*,†^	77.6 (59.3–99.7)	─

*Note*: Data are expressed as mean ± SD, median (IQR) or *n* (%).

Abbreviations: BMI, body mass index; FEF, forced expiratory flow; FEV1, forced expiratory volume in the first second; FVC, forced vital capacity; LCQ, Leicester Cough Questionnaire; MMEF, maximal mid‐expiratory flow; VAS, visual analogue scale.

* *p* < 0.05; compared with the HC group. ##*p* < 0.01; compared with the PIC group. †*p* < 0.05; †††*p* < 0.001; compared with the PIC group. Otherwise, nonsignificant (*p* > 0.05) between different groups.

While sputum lymphocyte percentages showed no significant differences between healthy individuals and PICC (or PISC) groups, the R‐PISC group exhibited markedly reduced lymphocyte proportions compared to PISC patients. FEF75 (% predicted) was significantly reduced in PICC patients compared to both healthy controls and PISC patients.

### 2.2 Cough Sensitivity and Airway Inflammation

**FIGURE 1:**
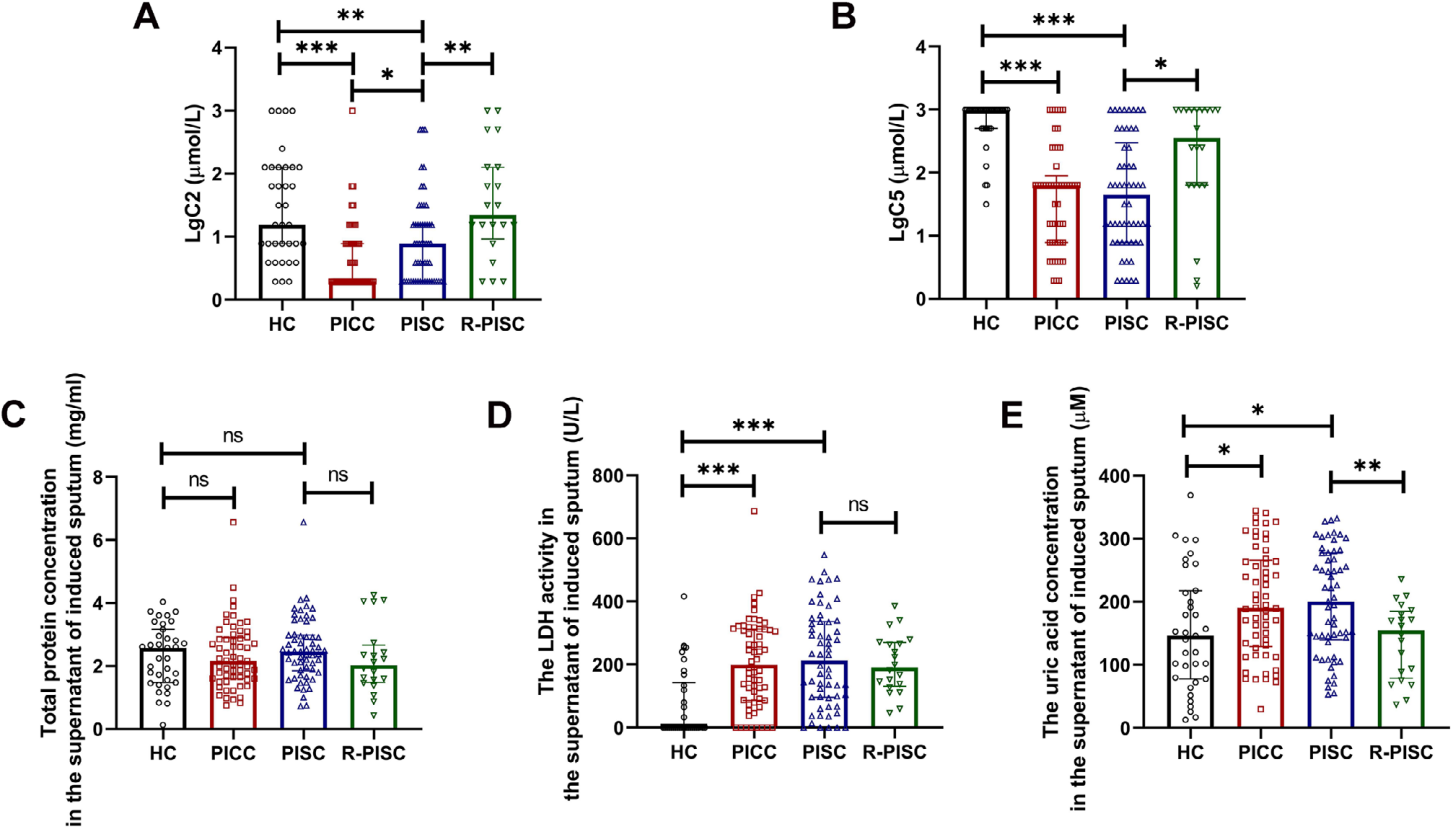
LgC2 (A) and LgC5 (B) among the HC group (*n* = 35), the PICC group (*n* = 53), the PISC group (*n* = 54), and the R‐PISC group (*n* = 20). Total protein concentration (C), LDH activity (D), and uric acid concentration (E) in the supernatant of induced sputum among the HC group (*n* = 35), the PICC group (*n* = 57), the PISC group (*n* = 57), and the R‐PISC group (*n* = 20). Data are shown as median (IQR). *p < 0.05; **p < 0.01; ***p < 0.001. C2, the lowest concentration of capsaicin that caused two or more coughs; C5, the lowest concentration of capsaicin that caused five or more coughs, HC, healthy controls, LDH, lactic dehydrogenase; ns, nonsignificant; PICC, postinfectious chronic cough; PISC, postinfectious subacute cough; R‐PISC, recovered individuals with postinfectious subacute cough.

LgC2 and LgC5 levels were significantly reduced in both PISC and PICC patients relative to healthy controls. R‐PISC subjects exhibited significantly higher levels of LgC2 and LgC5 compared to PISC patients. Cough sensitivity did not differ significantly between healthy controls and R‐PISC subjects. Significantly decreased LgC2 was noted in PICC versus PISC patients (Figure 1A,B). Lactate dehydrogenase (LDH) activity and uric acid concentration in sputa represent two biomarkers for assessing airway inflammation [32, 33]. LDH, a cytoplasmic enzyme in numerous cell types, is released into extracellular spaces following cellular lysis. Uric acid, derived from purine metabolism, is chiefly produced by airway epithelial cells. Sputum LDH activity and uric acid concentration were significantly higher in PISC and PICC patients than in healthy controls. R‐PISC subjects demonstrated significantly lower uric acid concentrations compared to PISC patients (Figure 1D,E).

### 2.3 Analyses of Blood Lymphocytes and Cytokines

**FIGURE 2:**
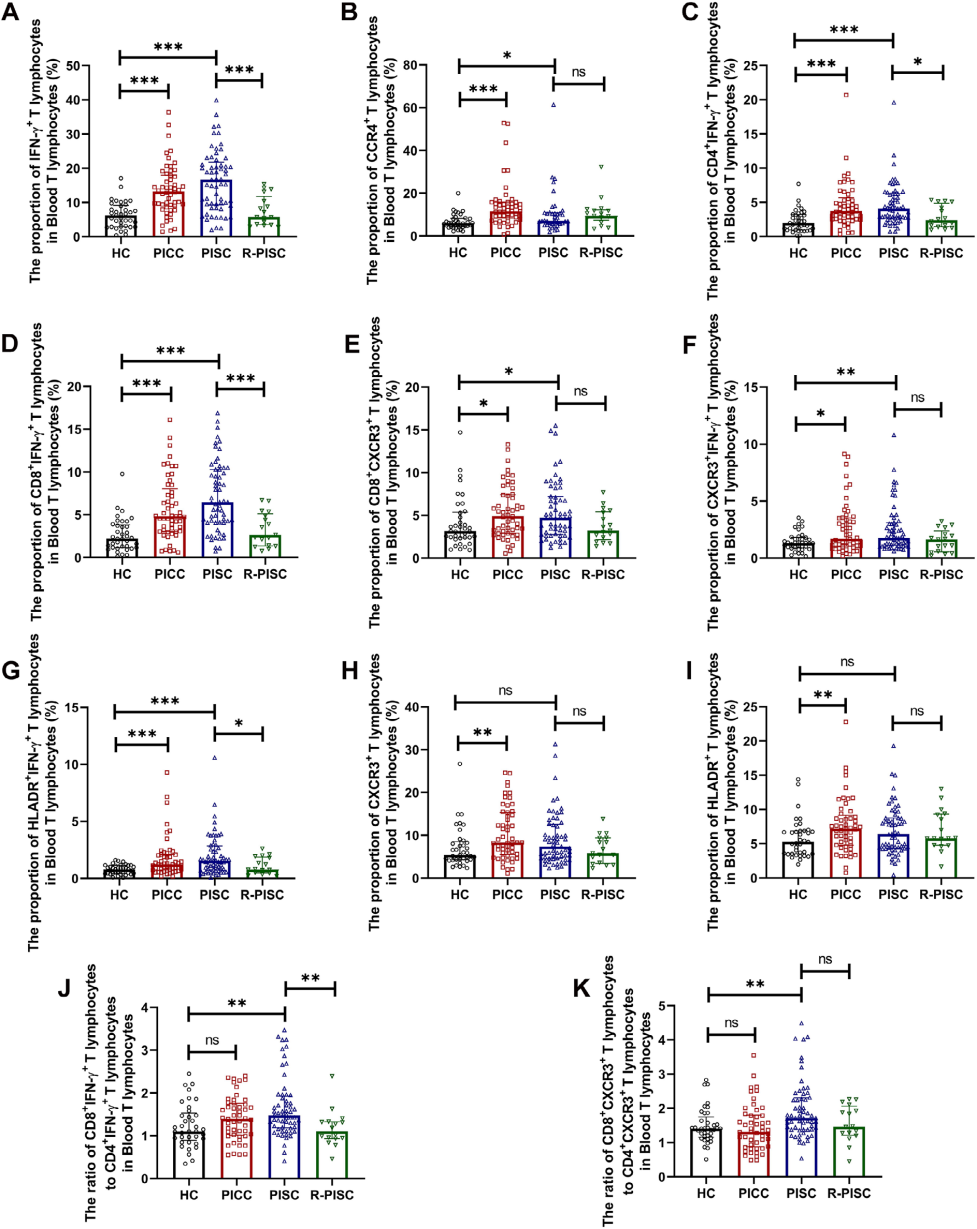
Flow cytometric analyses of T‐lymphocyte sub‐populations in blood T lymphocytes among the HC group, the PICC group, the PISC group, and the R‐PISC group. (A) The proportion of IFN‐γ^+^ T lymphocytes in blood T lymphocytes. (B) The proportion of CCR4^+^ T lymphocytes in blood T lymphocytes. (C) The proportion of CD4^+^IFN‐γ^+^ T lymphocytes in blood T lymphocytes. (D) The proportion of CD8^+^IFN‐γ^+^ T lymphocytes in blood T lymphocytes. (E) The proportion of CD8^+^CXCR3^+^ T lymphocytes in blood T lymphocytes. (F) The proportion of CXCR3^+^IFN‐γ^+^ T lymphocytes in blood T lymphocytes. (G) The proportion of HLADR^+^IFN‐γ^+^ T lymphocytes in blood T lymphocytes. (H) The proportion of CXCR3^+^ T lymphocytes in blood T lymphocytes. (I) The proportion of HLADR^+^ T lymphocytes in blood T lymphocytes. (J) The ratio of CD8^+^IFN‐γ^+^ T lymphocytes to CD4^+^IFN‐γ^+^ T lymphocytes in blood T lymphocytes. (K) The ratio of CD8^+^CXCR3^+^ T lymphocytes to CD4^+^CXCR3^+^ T lymphocytes in blood T lymphocytes. Data are shown as median (IQR). Each point on the graphs represents a sample. *p < 0.05; **p < 0.01; ***p < 0.001. CCR4, CC chemokine receptor 4; CD, cluster of differentiation; CXCR3, CXC chemokine receptor 3; HC, healthy controls; HLADR, human leukocyte antigen DR; IFN, interferon; ns, nonsignificant; PICC, postinfectious chronic cough; PISC, postinfectious subacute cough; R‐PISC, recovered individuals with postinfectious subacute cough.

Compared to healthy controls, both PICC and PISC patients exhibited significantly elevated proportions of IFN‐γ^+^ T lymphocytes, CCR4^+^ T lymphocytes (Th2 lymphocytes), CD4^+^IFN‐γ^+^ T lymphocytes (Th1 lymphocytes), CD8^+^IFN‐γ^+^ T lymphocytes, CD8^+^CXCR3^+^ T lymphocytes, CXCR3^+^IFN‐γ^+^ T lymphocytes, and HLADR^+^IFN‐γ^+^ T lymphocytes in blood. R‐PISC subjects exhibited significantly lower proportions of IFN‐γ^+^ T lymphocytes, Th1 lymphocytes, CD8^+^IFN‐γ^+^ T lymphocytes, and HLADR^+^IFN‐γ^+^ T lymphocytes in blood compared to PISC patients (Figure 2A–G). Consistent with their chemotactic response to 1000 ng/mL IP‐10 (Table S2), PICC but not PISC patients exhibited significantly higher proportions of CXCR3^+^ (activated) T lymphocytes and HLADR^+^ (activated) T lymphocytes in blood compared to healthy controls (Figure 2H,I). Plasma IFN‐γ and IL‐1β concentrations were markedly increased in PISC patients relative to healthy controls. The R‐PISC group demonstrated significantly lower plasma concentrations of IFN‐γ, IL‐1β, and IL‐6 compared to the PISC group (Table S3).

### 2.4 Analyses of Lymphocytes and Cytokines in Sputa

**FIGURE 3:**
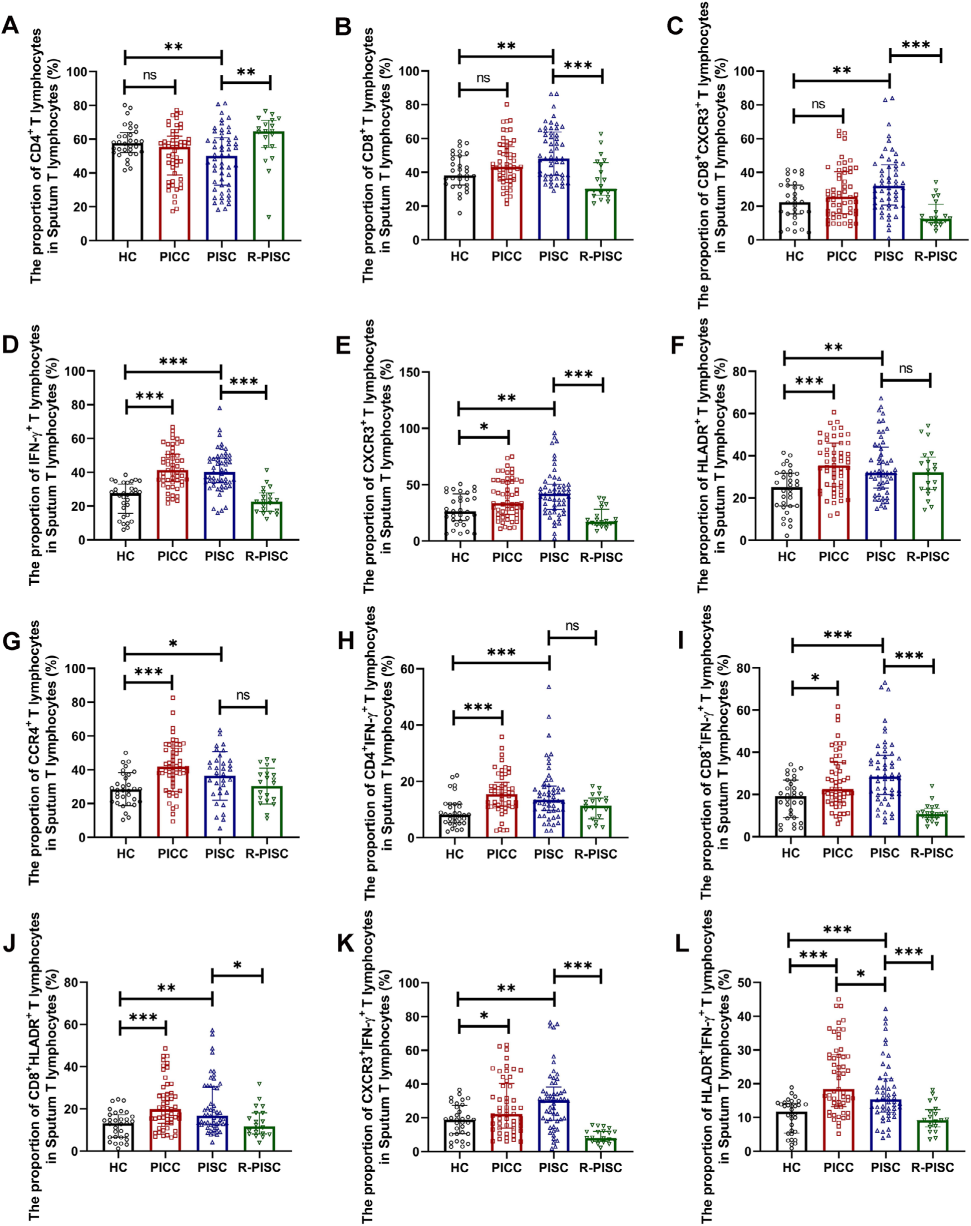
Flow cytometric analyses of T‐lymphocyte sub‐populations in sputum. Group data showing the proportion of CD4^+^ T lymphocytes (A), CD8^+^ T lymphocytes (B), CD8^+^CXCR3^+^ T lymphocytes (C), IFN‐γ^+^ T lymphocytes (D), CXCR3^+^ T lymphocytes (E), HLADR^+^ T lymphocytes (F), CCR4^+^ T lymphocytes (G), CD4^+^IFN‐γ^+^ T lymphocytes (H), CD8^+^IFN‐γ^+^ T lymphocytes (I), CD8^+^HLADR^+^ T lymphocytes (J), CXCR3^+^IFN‐γ^+^ T lymphocytes (K), and HLADR^+^IFN‐γ^+^ T lymphocytes (L) in sputum T lymphocytes among the HC group, the PICC group, the PISC group, and the R‐PISC group. Data are shown as mean ± SD in Figure G. Otherwise, data are shown as median (IQR). Each point on the graphs represents a sample. *p < 0.05; **p < 0.01; ***p < 0.001. CCR4, CC chemokine receptor 4; CD, cluster of differentiation; CXCR3, CXC chemokine receptor 3; HC healthy controls; HLADR, human leukocyte antigen DR; IFN, interferon; ns, nonsignificant; PICC, postinfectious chronic cough; PISC, postinfectious subacute cough; R‐PISC, recovered individuals with postinfectious subacute cough.

Compared to healthy controls, PISC but not PICC patients exhibited significantly altered T lymphocyte profiles in sputa, featuring (1) reduced CD4^+^ T‐lymphocyte proportions, (2) elevated CD8^+^ T‐lymphocyte proportions, and (3) increased CD8^+^CXCR3^+^ T‐lymphocyte proportions (Figure 3A–C). Compared with healthy controls, both PICC and PISC patients exhibited markedly elevated proportions of IFN‐γ^+^ T lymphocytes, CXCR3^+^ T lymphocytes, HLADR^+^ T lymphocytes, Th2 lymphocytes, Th1 lymphocytes, CD8^+^IFN‐γ^+^ T lymphocytes, CD8^+^HLADR^+^ T lymphocytes, CXCR3^+^IFN‐γ^+^ T lymphocytes, and HLADR^+^IFN‐γ^+^ T‐lymphocytes in sputa. R‐PISC subjects showed significantly lower proportions of CD8^+^ T lymphocytes, IFN‐γ^+^ T lymphocytes, CXCR3^+^ T lymphocytes, CD8^+^IFN‐γ^+^ T lymphocytes, CD8^+^HLADR^+^ T lymphocytes, CD8^+^CXCR3^+^ T lymphocytes, CXCR3^+^IFN‐γ^+^ T lymphocytes, and HLADR^+^IFN‐γ^+^ T lymphocytes in sputa compared to PISC patients (Figure 3D–L). PICC patients exhibited significantly higher proportions of HLADR^+^IFN‐γ^+^ T‐lymphocytes in sputum samples compared to PISC patients (Figure 3L). Both PICC and PISC patients exhibited significantly elevated ratios of CD8^+^/CD4^+^ T lymphocytes and CD8^+^HLADR^+^/CD4^+^HLADR^+^ T lymphocytes in sputa. These ratios were significantly reduced in R‐PISC subjects (Figure S1).

**FIGURE 4:**
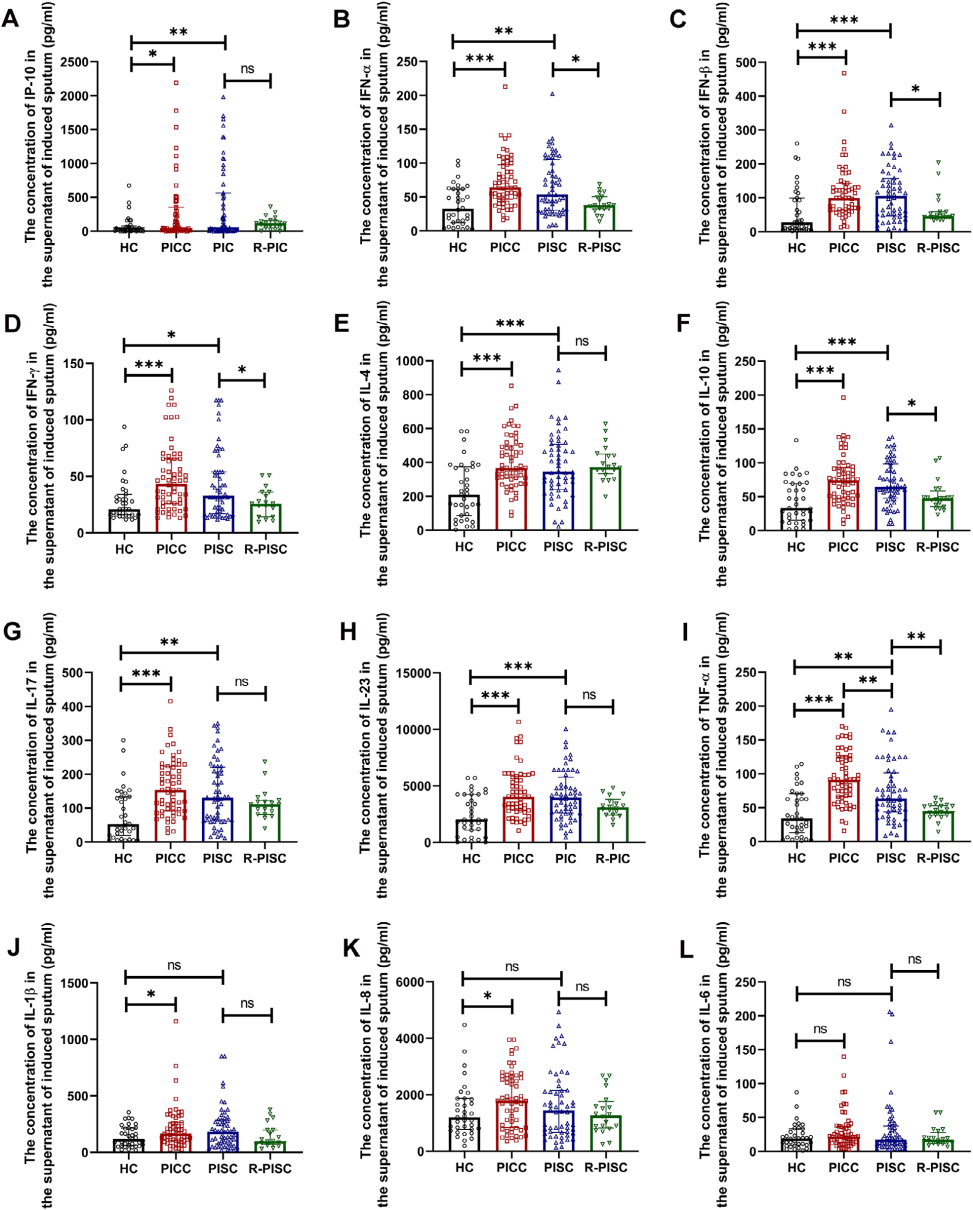
The concentration of cytokines in the supernatant of induced sputum among the HC group, the PICC group, the PISC group, and the R‐PISC group. (A) IP‐10. (B) IFN‐α. (C) IFN‐β. (D) IFN‐γ. (E) IL‐4. (F) IL‐10. (G) IL‐17. (H) IL‐23. (I) TNF‐α. (J) IL‐1β. (K) IL‐8. (L) IL‐6. Data are shown as median (IQR). Each point on the graphs represents a sample. *p < 0.05; **p < 0.01; ***p < 0.001. HC, healthy controls; IFN, interferon; IL, interleukin; IP, interferon‐γ‐inducible protein; PICC, postinfectious chronic cough; ns, nonsignificant; PISC, postinfectious subacute cough; R‐PISC, recovered individuals with postinfectious subacute cough; TNF, tumor necrosis factor.

Both PICC and PISC patients exhibited significantly elevated concentrations of IP‐10, IFN‐α, IFN‐β, IFN‐γ, IL‐4, IL‐10, IL‐17, IL‐23, and TNF‐α in sputa compared to healthy controls. R‐PISC subjects displayed significantly lower levels of multiple cytokines in sputa versus PISC patients, including IFN‐α, IFN‐β, IFN‐γ, IL‐10, and TNF‐α (Figure 4A–I). PICC patients exhibited significantly higher sputum concentrations of TNF‐α compared to PISC patients (Figure 4I). PICC but not PISC patients demonstrated significantly elevated sputum concentrations of IL‐1β and IL‐8 compared to healthy controls (Figure 4J,K).

### 2.5 Cough Sensitivity and Airway Inflammatory Marker Were Correlated With Both Activated IFN‐γ^+^ T‐lymphocytes and Cytokines in Sputum Samples

Despite potential confounding by individual clinical variability, significant negative correlations were maintained between LgC5 and peripheral blood proportions of IFN‐γ^+^ T lymphocytes, CD8^+^IFN‐γ^+^ T lymphocytes, and activated IFN‐γ^+^ T lymphocytes (Table S4), indicating that heightened capsaicin cough sensitivity correlates with systemic IFN‐γ‐producing T lymphocytes.

**FIGURE 5:**
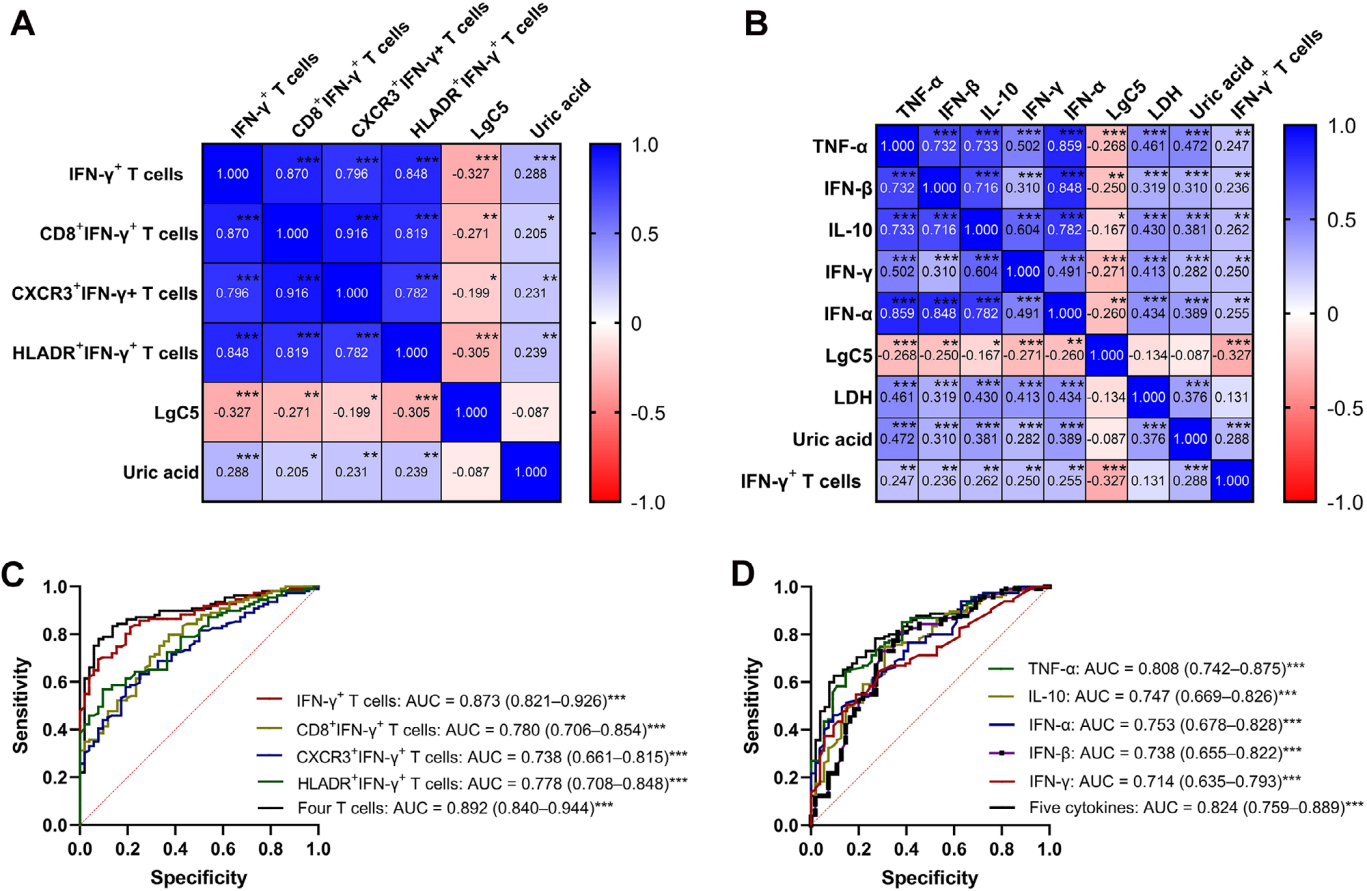
The correlation matrix and receiver operating characteristic curves of cough risk prediction models. (A) Correlations of sputum T‐lymphocyte sub‐populations with LgC5 and uric acid concentration in sputa. (B) Correlations of sputum cytokine concentrations with LgC5, LDH activity, uric acid concentration, and IFN‐γ^+^ T lymphocytes in sputa. The Spearman's *r* value is presented in each square. (C) Area under the curve of single and combined sputum T‐lymphocyte sub‐populations shows the performance of logistic regression models. (D) Area under the curve of single and combined sputum cytokines shows the performance of logistic regression models. *n* = 162. *p < 0.05; **p < 0.01; ***p < 0.001. C5, the lowest concentration of capsaicin that caused five or more coughs; CD, cluster of differentiation; CXCR3, CXC chemokine receptor 3; HLADR, human leukocyte antigen DR; IFN, interferon; IL, interleukin; LDH, lactic dehydrogenase; ns, nonsignificant;TNF, tumor necrosis factor.

As shown in Figure 5A, LgC5 demonstrated significant negative correlations with IFN‐γ^+^ T lymphocytes, CD8^+^IFN‐γ^+^ T lymphocytes, CXCR3^+^IFN‐γ^+^ T lymphocytes, and HLADR^+^IFN‐γ^+^ T lymphocytes in sputum samples. Moreover, uric acid concentration showed significant positive correlations with these T lymphocyte subsets in sputum samples.

LgC5 demonstrated significant negative correlations with sputum concentrations of IFN‐γ, TNF‐α, IFN‐β, IL‐10, and IFN‐α. Both sputum LDH activity and uric acid concentration showed significant positive correlations with these cytokine concentrations in sputum samples. Sputum IFN‐γ^+^ T lymphocytes were positively correlated with these cytokine concentrations in sputa (Figure 5B).

### 2.6 Construction of Cough Risk Prediction Model Based on Sputum T‐Lymphocyte Sub‐Populations and Sputum Cytokines

Based on significant correlations between cough sensitivity and both sputum T‐lymphocyte sub‐populations and sputum cytokines, logistic regression models were constructed to predict cough risk factors for patients with postinfectious cough. All four sputum T‐lymphocyte sub‐populations (IFN‐γ^+^ T lymphocytes, CD8^+^IFN‐γ^+^ T lymphocytes, CXCR3^+^IFN‐γ^+^ T lymphocytes, and HLADR^+^IFN‐γ^+^ T lymphocytes) alone showed an area under the curve (AUC) of more than 0.7, and the combination of them reached the highest AUC of 0.892 for the prediction of cough risk (Figure 5C). All five sputum cytokines (IFN‐γ, TNF‐α, IFN‐β, IL‐10, and IFN‐α) alone showed an AUC of more than 0.7, and the combination of them reached the highest AUC of 0.824 for the prediction of cough risk (Figure 5D).

## 3 Discussion

In our study, both PICC and PISC patients exhibited markedly higher levels of activated IFN‐γ^+^ T lymphocytes in blood compared to controls. These patients also exhibited heightened cough sensitivity, elevated airway inflammatory markers, increased proportions of activated IFN‐γ^+^ T lymphocytes, and elevated cytokine concentrations in sputa. In R‐PISC subjects, activated IFN‐γ^+^ T lymphocytes, cough hypersensitivity, and airway inflammation were reduced, suggesting resolution of inflammatory responses with disease improvement. Cough sensitivity and airway inflammatory markers showed significant correlations with activated IFN‐γ^+^ T lymphocytes and cytokine levels in sputa. Logistic regression models identified these activated IFN‐γ^+^ T lymphocytes and cytokines in sputa as significant predictive biomarkers for cough risk. This study elucidates key immune mechanisms underlying postinfectious cough progression and resolution, offering clinically relevant insights into how inflammatory signatures may inform targeted treatment strategies.

For the first time, our results show that both PICC and PISC patients exhibited significantly elevated proportions of IFN‐γ^+^ T lymphocytes, CD8^+^IFN‐γ^+^ T lymphocytes, and activated IFN‐γ^+^ T lymphocytes in blood, all of which normalized in R‐PISC subjects. LgC5 was inversely associated with proportions of these T‐lymphocyte subsets in blood. These results indicate that cough sensitivity might be positively associated with blood‐activated IFN‐γ^+^ T lymphocytes in PICC and PISC patients. Similarly, chronic cough in asthma patients has been associated with elevated systemic inflammatory biomarkers in blood [34]. Blood levels of activated CD8^+^ T lymphocytes and CD8^+^IFN‐γ^+^ T lymphocytes are increased in patients with prolonged coughing [35, 36]. Blood T lymphocytes serve as a reservoir for airway T‐lymphocyte recruitment, with enhanced trafficking during inflammatory conditions [37]. We have demonstrated that blood‐derived activated IFN‐γ^+^ T lymphocytes migrate to the airways in mice, directly contributing to lymphocytic airway inflammation [38].

Sputum composition analysis represents an established technique for characterizing and quantifying airway inflammation [39]. Our results suggest that both PICC and PISC patients had elevated levels of cough sensitivity, inflammatory marker of uric acid, activated T lymphocytes, IFN‐γ^+^ T lymphocytes, CD8^+^/CD4^+^ T lymphocyte ratios in sputa, as well as elevated concentrations of IFN‐γ, TNF‐α, IFN‐α, IFN‐β, and IL‐10 in sputa, all of which were reduced in R‐PISC subjects. Cough sensitivity and airway inflammatory marker were associated with IFN‐γ^+^ T lymphocytes, CD8^+^IFN‐γ^+^ T lymphocytes, and activated IFN‐γ^+^ T lymphocytes in the airway. These elevated cytokines were correlated with cough sensitivity, inflammatory marker, and IFN‐γ^+^ T lymphocytes in the airway. Logistic regression identified these T‐lymphocyte subsets and cytokines in sputa as predictive biomarkers for cough risk. While sputum lymphocyte percentages showed no significant differences between healthy controls and PICC/PISC patients, we observed a marked reduction in R‐PISC subjects that paralleled clinical improvement in cough symptoms and airway inflammation. The substantial interindividual variability may explain the lack of significant differences in sputum lymphocyte percentages between HC and R‐PISC subjects. However, longitudinal analysis revealed a consistent decrease in sputum lymphocytes during recovery (R‐PISC), suggesting this parameter reflects disease resolution. While lymphocytes constitute only a minor cellular component in sputum, their percentage was significantly higher in bronchoalveolar lavage fluid (BALF) [40]. Lymphocytes emerge as the predominant cell population infiltrating the bronchial submucosa in both asthma and chronic bronchitis patients [40]. Following influenza viral infection, lymphocytes become the predominant cell type during the late‐phase in mouse BALF [13, 41]. The percentage of lymphocytes in BALF is elevated in patients with postinfectious cough [3]. Therefore, airway activated IFN‐γ^+^ T‐lymphocytes and dysregulated cytokines (especially IFN‐γ, TNF‐α, IFN‐α, IFN‐β, and IL‐10) might serve as inflammatory signatures in some patients with postinfectious cough. Similarly, sputum from chronic cough patients showed increased CD8^+^IFN‐γ^+^ T‐lymphocytes and Th1 lymphocytes [42]. Although sputum T‐lymphocyte counts showed no significant differences between idiopathic cough patients and normal subjects, lymphocyte counts in BALF are significantly elevated in idiopathic cough patients [43]. Chronic cough children show increased proportions of CD8^+^ T lymphocytes and CD8^+^CXCR3^+^ T lymphocytes in BALF [44]. Residual airway inflammation causes post‐virus persistent cough in patients [45]. Sputum LDH activity is increased in patients with infective acute asthma [32]. Airway cytokine levels constitute reliable biomarkers for severity assessment and outcome prediction in respiratory viral infections [46]. We have demonstrated that IFN‐γ can enhance cough sensitivity in both guinea pigs and human adults [24, 42]. Pulmonary IFN‐γ can also cause airway inflammation [19]. IFN‐α, IFN‐β, and TNF‐α were reported to induce cough hypersensitivity [25, 26]. IFN‐α, IFN‐β, and TNF‐α can also induce airway inflammation [47, 48]. The role of IL‐10 on airway inflammation is controversial [23]. Whether IL‐10 can directly enhance cough sensitivity remains uncertain. Further studies are needed to demonstrate whether activated IFN‐γ^+^ T lymphocytes serve as the primary cellular source of these elevated cytokines in the airway of PICC and PISC patients. Given our findings of T‐cell activation in postinfectious cough, immunosuppressive agents targeting T‐cell function (e.g., cyclosporine A) or IFN‐γ represent promising therapeutic candidates that warrant systematic clinical evaluation in randomized controlled trials.

The PICC phenotype was characterized by four key distinctions from PISC: (1) more severe clinical manifestations (worse LCQ scores and higher dry cough frequency), (2) greater small airway function (lower FEF75% of predicted), (3) higher levels of cough sensitivity (decreased LgC2), and (4) heightened airway inflammation (elevated activated IFN‐γ^+^ T‐lymphocytes and TNF‐α). Consistently, patients with chronic refractory cough demonstrated elevated sputum IFN‐γ^+^ T‐lymphocytes [42]. Sputum TNF‐α levels are increased in patients with chronic dry cough [49].

Our study has some limitations. First, although this study demonstrated the potential of airway activated IFN‐γ^+^ T lymphocytes and cytokines as inflammatory signatures for postinfectious cough, to what extent airway activated IFN‐γ^+^ T lymphocytes and cytokines are driving cough cannot be determined in this study. Second, this study's focus on postinfectious cough leaves open important questions about the specificity of the observed inflammatory signatures, as common pathways may exist across multiple cough‐related disorders. Third, this is a single‐center study. Enrolled subjects, came from South China, could not represent all populations around the world. Future multi‐center studies are needed to confirm a broader applicability. Fourth, the precise mechanistic origins of IFN‐γ^+^ T‐lymphocyte activation in postinfectious cough remain undefined, with the relative contributions of persistent viral antigen stimulation versus cytokine‐dependent bystander activation requiring further systematic investigation.

In conclusion, both PICC and PISC patients may have elevated levels of activated IFN‐γ^+^ T lymphocytes in blood. Postinfectious cough patients may exhibit airway inflammatory signatures characterized by activated IFN‐γ^+^ T lymphocytes and elevated levels of IFN‐γ, TNF‐α, IFN‐α, IFN‐β, and IL‐10. PICC patients may present with a more severe cough phenotype than PISC patients. This work provides the immunological foundation for developing targeted treatments for postinfectious cough.

## 4 Methods

All compounds and drugs are provided in Table S5.

### 4.1 Study Population

This prospective cohort study enrolled subjects from September 2019 to January 2024 in the First Affiliated Hospital of Guangzhou Medical University. Participants were recruited into four groups: (1) healthy controls (HC), (2) PICC patients, (3) PISC patients, and (4) R‐PISC subjects.

Healthy control participants (aged 18–70 years) met stringent inclusion criteria: (1) lifelong non‐smokers, (2) normal chest radiography, (3) absence of respiratory symptoms for ≥8 weeks, and (4) no history of chronic respiratory/allergic diseases. Pregnant or breast‐feeding women were also ineligible participants.

PICC patients who had no history of chronic cough presented with cough as a sole or predominant symptom lasting more than 8 weeks triggered by an acute respiratory tract infection. PISC patients who had no history of chronic cough presented with cough lasting 3–8 weeks triggered by an acute respiratory tract infection. If patient reported an abrupt onset of three or more of the following symptoms prior to a cough, such as nasal drainage, nasal obstruction, sneezing, sore throat and fever, a history of acute respiratory tract infection was considered, and the patient should not have those symptoms of acute respiratory tract infection seasonally. As described previously [50], PICC/PISC inclusion criteria required all of the following: (1) aged 18–70 years, (2) history of acute respiratory tract infection at the onset of cough, (3) cough protracted or worsening after the remission of acute symptoms, (4) irritating dry cough or cough with small amount of sputum, (5) normal spirometry without airway hyperresponsiveness, and (6) normal eosinophil percentage in induced sputum. The exclusion criteria were as follows: (1) current or former smokers, (2) pregnant or breast‐feeding women, (3) radiographic abnormalities, (4) pre‐existing chronic cough, (5) co‐morbidities such as asthma, chronic obstructive pulmonary disease, gastroesophageal reflux disease, upper airway cough syndrome, and other conditions that could contribute to sustaining the cough symptom, (6) allergic constitution, (7) complications of other serious respiratory diseases, and (8) taking angiotensin‐converting enzyme inhibitors.

All PISC patients received telephone follow‐ups for 8 weeks to document cough progression (to PICC) or resolution (R‐PISC). Patients who eventually developed into PICC patients were excluded from the PISC group. Some R‐PISC subjects were randomly invited to our hospital for subsequent tests. According to the volume of daily produced phlegm, cough phenotype was classified as dry cough (phlegm volume < 10 mL per day) or wet cough (phlegm volume > 10 mL per day). Demographic data of the four groups are detailed in Table 1. The sample sizes for the HC, PICC, PISC, and R‐PISC groups were determined based on similar studies [51, 52] and our preliminary experimental data on LgC5 and sputum IFN‐γ^+^ T lymphocytes.

All participants provided written informed consent before study participation. The written informed consent provides the participant with clear and accurate information about this study, including purpose, procedures, risks, and benefits. Protocols for biospecimen storage/use are compliant with the International Organization for Standardization (ISO) 20387:2018, and the international standard specifies general requirements for biobanks.

### 4.2 Procedures

**FIGURE 6:**
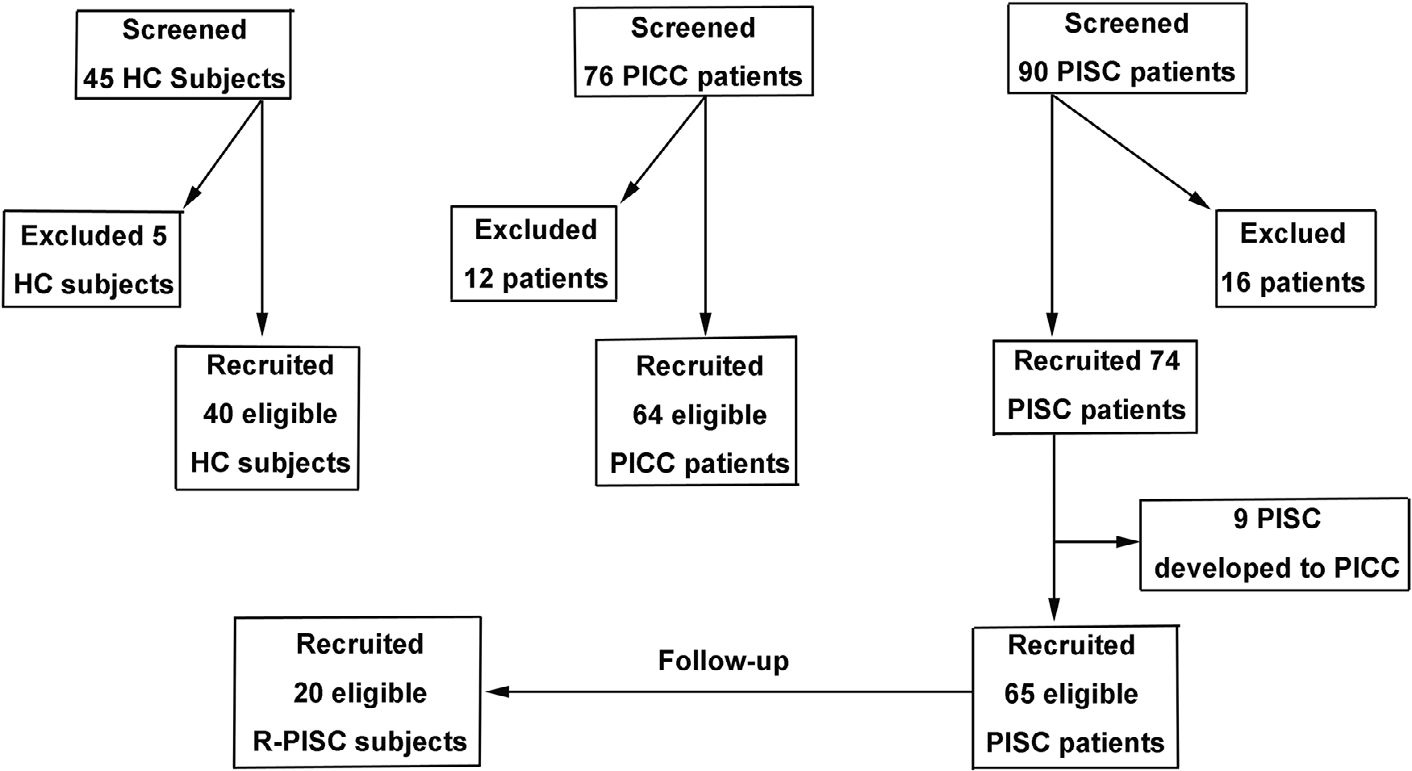
The inclusion process of the recruited subjects. HC, healthy controls; PICC, postinfectious chronic cough; PISC, postinfectious subacute cough; R‐PISC, recovered individuals with postinfectious subacute cough.

The enrolment process is shown in Figure 6. Forty‐five healthy controls were enrolled by advertisement. This study randomly recruited 90 PISC patients and 76 PICC patients who were referred to the outpatient clinic in the First Affiliated Hospital of Guangzhou Medical University. The initial assessment for the screening included medical history, a physical examination, vital signs, chest X‐ray, induced sputum test, routine blood test, spirometry test, and clinical questionnaire. After the screening assessment, we successfully recruited 74 PISC patients and 64 PICC patients. During longitudinal follow‐up, nine patients (12.2%) eventually developed into PICC patients, who were excluded from the PISC group. Twenty randomly selected subjects from the eligible PISC group, who had fully recovered (R‐PISC), were invited to our hospital for further testing. All experiments were performed and assessed by personnel blinded to group assignment. As described previously [42], cough reflex sensitivity was tested in all the enrolled subjects. Capsaicin cough challenge test was performed with a compressed air‐driven nebulizer controlled by a breath‐activated dosimeter (output, 0.025 mL per inhalation, Jaeger, Germany). Coughs were induced by progressively doubling concentrations of capsaicin solutions (1.95–1000 µmol/L) until five or more coughs were elicited. The cough threshold ‌C2‌ was defined as the lowest capsaicin concentration eliciting ‌two‌ or more coughs, and ‌C5‌ was defined as the lowest concentration eliciting ‌five‌ or more coughs. Finally, cough reflex sensitivity was presented as the base‐10 logarithm of C2 or C5 (LgC2 or LgC5). Cough symptoms were evaluated with the cough Visual Analogue Scale (VAS), Cough Symptom Score (CSS), and Leicester Cough Questionnaire (LCQ). The cough VAS was measured on a 100‐mm scale ranging from 0 to 100, with a higher score indicating a more severe cough. The CSS was a two‐part questionnaire scoring including daytime cough and night‐time cough, in which each part score ranges from 0 (no cough) to 5 (worst cough). The LCQ was a 19‐item cough‐specific health‐related quality‐of‐life questionnaire scoring from 3 to 21 (a lower score indicates worse cough‐specific life quality) [53].

Sputum was induced and subsequently processed following previously published procedures [42]. Briefly, sputum was induced using 3% hypertonic saline administered via an ultrasonic nebulizer. And then the induced sputum was weighted and mixed with 0.1% dithiothreitol at a volume four times that of the sputum. After filtration through a 48‐mm nylon mesh and centrifugation at 800 g for 5 min, the supernatant was collected and stored at −80°C for subsequent analysis (total protein concentration, uric acid concentration, lactic dehydrogenase activity, and cytokine concentrations). The pellet was resuspended with 2 mL phosphate‐buffered saline (PBS). Cell profiles were assessed through differential counting using hematoxylin‐eosin staining. Differential cell counts were performed on a minimum of 400 leukocytes to determine the percentages of neutrophils, macrophages, lymphocytes, and eosinophils in the induced sputum. The residual sputum cell suspension was processed for flow cytometry analysis.

A routine blood test was performed to determine the percentages of neutrophils, lymphocytes, and eosinophils in peripheral blood from all enrolled subjects. Additionally, 10 mL of blood was collected into anticoagulant tubes (BD, USA). After the centrifugation of blood at 1200 *g* for 5 min, the supernatant was extracted and stored at −80°C for cytokine analysis, while the pellet was resuspended in an equal volume of D‐Hank's solution. The blood cell suspension underwent both flow cytometric characterization and lymphocyte chemotaxis assays.

### 4.3 Flow Cytometry Analyses of Human Mononuclear Cells

As previously described [19], mononuclear cells were isolated from both sputum and blood cell suspension using density gradient centrifugation. Following centrifugation of isolated mononuclear cells, residual erythrocytes in the pellet were lysed using erythrocyte lysis buffer. Both sputum‐derived and peripheral blood mononuclear cells were resuspended in PBS for flow cytometric analysis. A portion of the isolated peripheral blood mononuclear cells was additionally used for transwell migration assays.

For immunostaining, cells were first stimulated with Golgiplug. After washing with PBS, cells were incubated with human Fc receptor blocking solution. Cells were then stained with a cocktail of CD3, CD4, CD8, CXCR3, HLADR, and CCR4 antibodies. Following surface staining, cells were washed fixed/permeabilized with Cytofix/Cytoperm. After additional washing, cells were blocked with human Fc receptor blocking reagent and then stained with an intracellular antibody (IFN‐γ). All flow cytometric analyses were performed using a BD FACSVerse flow cytometer (BD Biosciences, USA). Data analysis was conducted with FlowJo software (TreeStar, Ashland, OR). The gating strategy for flow cytometric analysis is detailed in Figures S2 and S3.

### 4.4 Cytokine Measurement in the Supernatant of Induced Sputum and Plasma

Cytokine concentrations in the supernatant of induced sputum and plasma were quantified using a human cytokine magnetic bead panel (LXSAHM‐12, RD) on the Luminex‐200 platform, following the manufacturer's protocol. Data analysis was performed with the Milliplex Analyst Software. The 12 detected cytokines included IP‐10, IFN‐α, IFN‐β, IFN‐γ, IL‐1β, IL‐4, IL‐6, IL‐8, IL‐10, IL‐17, IL‐23, and TNF‐α.

### 4.5 Transwell Migration Assays of Blood Lymphocytes

Lymphocyte migration was assessed in vitro using transwell chambers (5 µm; Corning Costar). At first, the extracted PBMC were resuspended in RPMI 1640 medium supplemented with 0.1% bovine serum albumin and seeded into the upper chambers. The lower chambers were added with the same medium supplemented with gradient concentrations of IP‐10 (0, 40, 200, and 1000 ng/mL). Following 4‐h incubation at 37°C in a 5% CO_2_ incubator, migrated lymphocytes in the lower chambers were quantified by flow cytometry using BD FACS VerseTM. The chemotactic index was calculated as the number of migrated lymphocytes in test condition divided by the number of migrated lymphocytes in the negative control without IP‐10.

### 4.6 Statistical Analysis

Data normality was evaluated using Shapiro–Wilk and Kolmogorov–Smirnov tests. Normally distributed data were presented as mean ± SD, whereas non‐normally distributed data were reported as median (IQR). For normally distributed data, intergroup differences were assessed using either (1) one‐way ANOVA with Bonferroni post hoc tests (for multiple comparisons) or (2) unpaired two‐tailed Student's *t*‐tests (for pairwise comparisons). For post hoc tests, Bonferroni's multiple comparison tests were run only if *F* achieved *p* < 0.05 and there was no significant variance inhomogeneity. Where data were not normally distributed, Kruskal–Wallis tests followed by Dunn's test or Mann–Whitney tests were applied to assess the difference between groups. Categorical variables were presented as numbers (%) and were compared using either chi‐square tests or Fisher's exact texts. Correlation analyses were assessed using Spearman's rank correlation tests. Logistic regression models were developed to identify significant cough risk factors for postinfectious cough. The predictive performance of these models was evaluated using receiver operating characteristic (ROC) curve analysis, with the AUC quantifying discrimination accuracy. Values of *p* < 0.05 were considered statistically significant.

## Author Contributions

Conception and design: Kefang Lai and Zheng Deng. Performing experiments: Zheng Deng, Tongtong Song, Wenbin Ding, Wei Luo, and Haodong Wu. Analysis and interpretation: Zheng Deng, Kefang Lai, Tongtong Song, and Wenbin Ding. Manuscript writing and revising: Zheng Deng, Kefang Lai, Tongtong Song, Wenbin Ding, Wei Luo, Jiaxing Xie, Haodong Wu, and Nanshan Zhong. Kefang Lai and Zheng Deng had directly accessed and verified all of the data. All authors have read and approved the final manuscript.

## Ethics Statement

All subjects provided written informed consent for this study, which was approved by the Ethics Committee of the First Affiliated Hospital of Guangzhou Medical University (Approval number: 2019 No. 77).

## Conflicts of Interest

The authors declare no conflicts of interests.

## Supporting information




**Figure S1**: Flow cytometric analyses of sputum T lymphocyte sub‐populations among the HC group, the PICC group, the PISC group and the R‐PISC group. (A) The ratio of CD8^+^ T lymphocytes to CD4^+^ T lymphocytes in sputum T lymphocytes. (B) The ratio of CD8^+^HLADR^+^ T lymphocytes to CD4^+^HLADR^+^ T lymphocytes in sputum T lymphocytes. Data are shown as median (IQR). Each point on the graphs represents a sample. *p < 0.05; **p < 0.01; ***p < 0.001. CD = cluster of differentiation. HLADR = human leukocyte antigen DR.
**Figure S2**: Multigating strategy for the analysis of surface antigens and intracellular cytokines of peripheral blood mononuclear cells. Lymphocytes (R1) were identified based on gating of forward scatter area (FSC‐A) and side scatter area (SSC‐A). And then forward scatter height (FSC‐H) and FSC‐A gating was used to obtain single lymphocytes (R2). Positive staining for CD3 was then used to distinguish T lymphocytes (R3). Subsequently, representative plots showed percentage of CD4^+^ T lymphocytes (R4), CD8^+^ T lymphocytes (R5), CXCR3^+^ T lymphocytes (R6), HLADR^+^ T lymphocytes (R7), IFN‐γ^+^ T lymphocytes (R8), CCR4^+^ T lymphocytes (R9), CD4^+^CXCR3^+^ T lymphocytes (R10), CD8^+^CXCR3^+^ T lymphocytes (R11), CD4^+^IFN‐γ^+^ T lymphocytes (R12), CD8^+^IFN‐γ^+^ T lymphocytes (R13), CXCR3^+^IFN‐γ^+^ T lymphocytes (R14), and HLADR^+^IFN‐γ^+^ T lymphocytes (R15) within all the T lymphocytes.
**Figure S3**: Multigating strategy for the analysis of surface antigens and intracellular cytokines of sputum mononuclear cells. Lymphocytes (R1) were identified based on gating of forward scatter area (FSC‐A) and side scatter area (SSC‐A). And then forward scatter height (FSC‐H) and FSC‐A gating was used to obtain single lymphocytes (R2). Positive staining for CD3 was then used to distinguish T lymphocytes (R3). Subsequently, representative plots showed percentage of CD4^+^ T lymphocytes (R4), CD8^+^ T lymphocytes (R5), CXCR3^+^ T lymphocytes (R6), HLADR^+^ T lymphocytes (R7), IFN‐γ^+^ T lymphocytes (R8), CCR4^+^ T lymphocytes (R9), CD4^+^CXCR3^+^ T lymphocytes (R10), CD8^+^CXCR3^+^ T lymphocytes (R11), CD4^+^IFN‐γ^+^ T lymphocytes (R12), CD8^+^IFN‐γ^+^ T lymphocytes (R13), CD4^+^HLADR^+^ T lymphocytes (R14), CD8^+^HLADR^+^ T lymphocytes (R15), CXCR3^+^IFN‐γ^+^ T lymphocytes (R16), and HLADR^+^IFN‐γ^+^ T lymphocytes (R17) within all the T lymphocytes.
**Table S1**: Demographics and clinical characteristics of PISC and R‐PISC at baseline.
**Table S2**: IP‐10‐induced migration of blood lymphocytes in vitro.
**Table S3**: Cytokine concentrations in plasma.
**Table S4**: Correlations of blood T lymphocyte sub‐populations with LgC5.
**Table S5**: Compounds and materials used in this study.

## Data Availability

The data that support the findings of this study are available from the corresponding author on reasonable request.
