# Supplementary material for: Activated Interferon‐γ‐Positive T Lymphocytes and Cytokine Signatures in Patients With Postinfectious Cough
Source: MedComm (2020). 2025 Aug 24;6(9):e70340. doi: 10.1002/mco2.70340 (PMC12375690; doi:10.1002/mco2.70340)
Supplement: Supplementary file 1 — Figure S1: Flow cytometric analyses of sputum T lymphocyte sub‐populations among the HC group, the PICC group, the PISC group and the R‐PISC group. (A) The ratio of CD8+ T lymphocytes to CD4+ T lymphocytes in sputum T lymphocytes. (B) The ratio of CD8+HLADR+ T lymphocytes to CD4+HLADR+ T lymphocytes in sputum T lymphocytes. Data are shown as median (IQR). Each point on the graphs represents a sample. *p < 0.05; **p < 0.01; ***p < 0.001. CD = cluster of differentiation. HLADR = human leukocyte antigen DR. Figure S2: Multigating strategy for the analysis of surface antigens and intracellular cytokines of peripheral blood mononuclear cells. Lymphocytes (R1) were identified based on gating of forward scatter area (FSC‐A) and side scatter area (SSC‐A). And then forward scatter height (FSC‐H) and FSC‐A gating was used to obtain single lymphocytes (R2). Positive staining for CD3 was then used to distinguish T lymphocytes (R3). Subsequently, representative plots showed percentage of CD4+ T lymphocytes (R4), CD8+ T lymphocytes (R5), CXCR3+ T lymphocytes (R6), HLADR+ T lymphocytes (R7), IFN‐γ+ T lymphocytes (R8), CCR4+ T lymphocytes (R9), CD4+CXCR3+ T lymphocytes (R10), CD8+CXCR3+ T lymphocytes (R11), CD4+IFN‐γ+ T lymphocytes (R12), CD8+IFN‐γ+ T lymphocytes (R13), CXCR3+IFN‐γ+ T lymphocytes (R14), and HLADR+IFN‐γ+ T lymphocytes (R15) within all the T lymphocytes. Figure S3: Multigating strategy for the analysis of surface antigens and intracellular cytokines of sputum mononuclear cells. Lymphocytes (R1) were identified based on gating of forward scatter area (FSC‐A) and side scatter area (SSC‐A). And then forward scatter height (FSC‐H) and FSC‐A gating was used to obtain single lymphocytes (R2). Positive staining for CD3 was then used to distinguish T lymphocytes (R3). Subsequently, representative plots showed percentage of CD4+ T lymphocytes (R4), CD8+ T lymphocytes (R5), CXCR3+ T lymphocytes (R6), HLADR+ T lymphocytes (R7), IFN‐γ+ T lymphocytes (R8), CCR4+ T lymphocytes (R9 [file MCO2-6-e70340-s001.docx]

**Supplementary Information**

**Activated interferon-γ-positive T lymphocytes and cytokine signatures in patients with postinfectious cough**

Zheng Deng^1,2,#^, Tongtong Song^1,#^, Wenbin Ding^1,#^, Wei Luo^1^, Jiaxing Xie^1^, Haodong Wu^1^, Nanshan Zhong^1,2^, and Kefang Lai^1,2^*

^1^State Key Laboratory of Respiratory Disease, Guangzhou Institute of Respiratory Disease, The First Affiliated Hospital of Guangzhou Medical University, Guangzhou, China.

^2^Guangzhou National Laboratory, Guangzhou, China.

**^#^**These three authors contributed equally to the research and are considered co-first authors of the publication.

* Correspondence and requests for reprints should be addressed to Kefang Lai, PhD, State Key Laboratory of Respiratory Disease, Guangzhou Institute of Respiratory Disease, The First Affiliated Hospital of Guangzhou Medical University, Guangzhou, China. Tel: +86 20 8156 6841; Fax: +86 20 8156 6841. E-mail: klai@163.com

**Supplementary Figures and Figure legends**


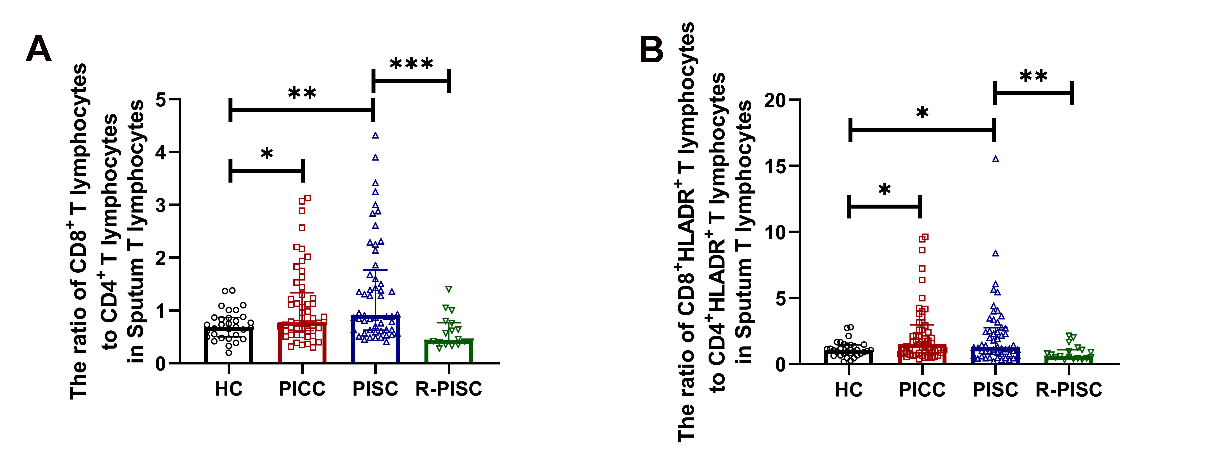


**Figure S1.** Flow cytometric analyses of sputum T lymphocyte sub-populations among the HC group, the PICC group, the PISC group and the R-PISC group. (A) The ratio of CD8^+^ T lymphocytes to CD4^+^ T lymphocytes in sputum T lymphocytes. (B) The ratio of CD8^+^HLADR^+^ T lymphocytes to CD4^+^HLADR^+^ T lymphocytes in sputum T lymphocytes. Data are shown as median (IQR). Each point on the graphs represents a sample. **P* < 0.05; ***P* < 0.01; ****P* < 0.001. CD = cluster of differentiation. HLADR = human leukocyte antigen DR.


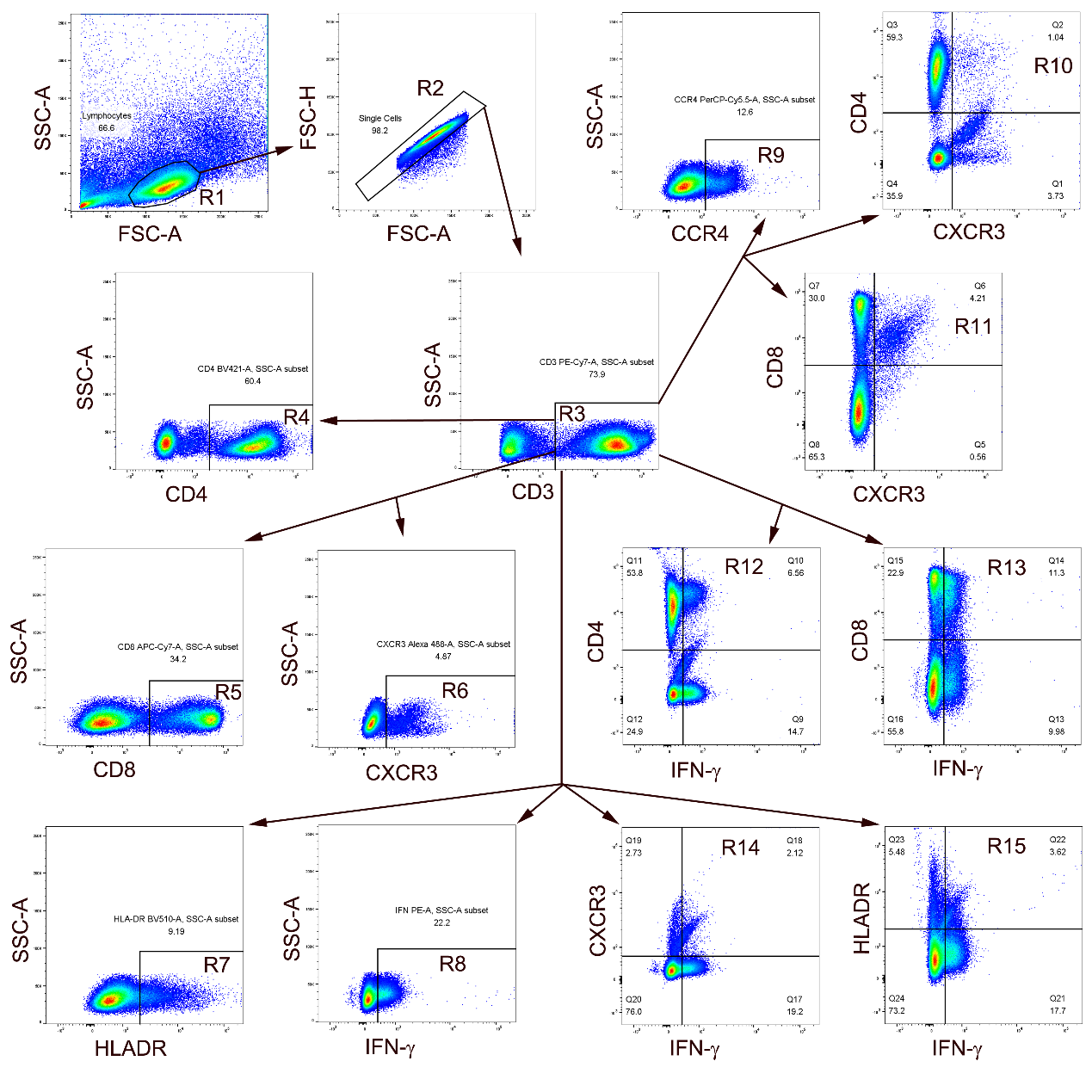


**Figure S2.** Multigating strategy for the analysis of surface antigens and intracellular cytokines of peripheral blood mononuclear cells. Lymphocytes (R1) were identified based on gating of forward scatter area (FSC-A) and side scatter area (SSC-A). And then forward scatter height (FSC-H) and FSC-A gating was used to obtain single lymphocytes (R2). Positive staining for CD3 was then used to distinguish T lymphocytes (R3). Subsequently, representative plots showed percentage of CD4^+^ T lymphocytes (R4), CD8^+^ T lymphocytes (R5), CXCR3^+^ T lymphocytes (R6), HLADR^+^ T lymphocytes (R7), IFN-γ^+^ T lymphocytes (R8), CCR4^+^ T lymphocytes (R9), CD4^+^CXCR3^+^ T lymphocytes (R10), CD8^+^CXCR3^+^ T lymphocytes (R11), CD4^+^IFN-γ^+^ T lymphocytes (R12), CD8^+^IFN-γ^+^ T lymphocytes (R13), CXCR3^+^IFN-γ^+^ T lymphocytes (R14), and HLADR^+^IFN-γ^+^ T lymphocytes (R15) within all the T lymphocytes.


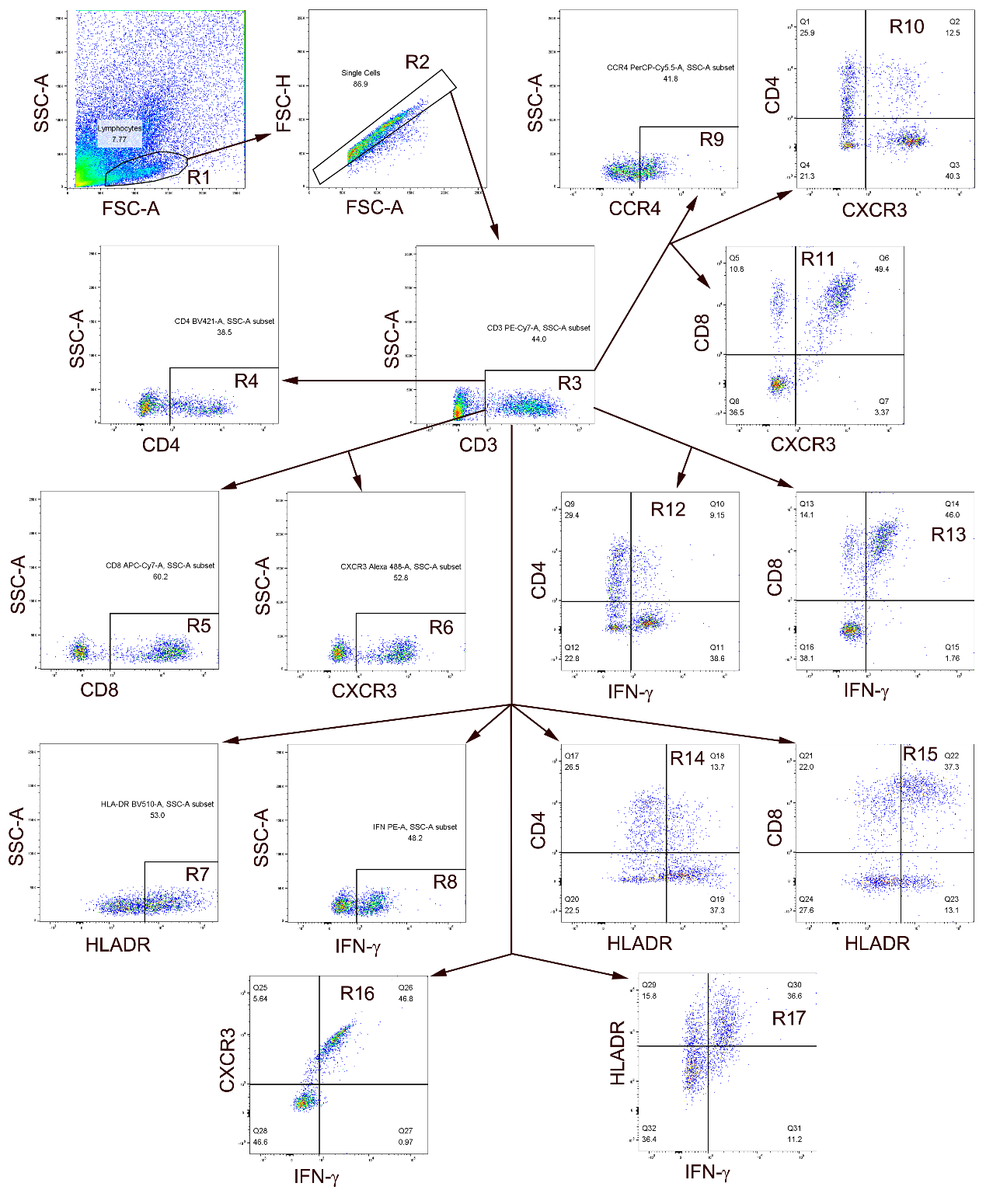


**Figure S3.** Multigating strategy for the analysis of surface antigens and intracellular cytokines of sputum mononuclear cells. Lymphocytes (R1) were identified based on gating of forward scatter area (FSC-A) and side scatter area (SSC-A). And then forward scatter height (FSC-H) and FSC-A gating was used to obtain single lymphocytes (R2). Positive staining for CD3 was then used to distinguish T lymphocytes (R3). Subsequently, representative plots showed percentage of CD4^+^ T lymphocytes (R4), CD8^+^ T lymphocytes (R5), CXCR3^+^ T lymphocytes (R6), HLADR^+^ T lymphocytes (R7), IFN-γ^+^ T lymphocytes (R8), CCR4^+^ T lymphocytes (R9), CD4^+^CXCR3^+^ T lymphocytes (R10), CD8^+^CXCR3^+^ T lymphocytes (R11), CD4^+^IFN-γ^+^ T lymphocytes (R12), CD8^+^IFN-γ^+^ T lymphocytes (R13), CD4^+^HLADR^+^ T lymphocytes (R14), CD8^+^HLADR^+^ T lymphocytes (R15), CXCR3^+^IFN-γ^+^ T lymphocytes (R16), and HLADR^+^IFN-γ^+^ T lymphocytes (R17) within all the T lymphocytes.

**Supplementary Tables**

**Table S1. Demographics and clinical characteristics of PISC and R-PISC at baseline**

|  | PISC | R-PISC at baseline |
| --- | --- | --- |
| Total number of subjects | 65 | 20 |
| Age (years) | 36.0 (31.0-45.0) | 34.5 (31.3-40.8) |
| Female | 42 (64.6%) | 12 (60%) |
| BMI (Kg/m^2^) | 22.0 (20.0-24.2) | 22.0 (20.0-24.8) |
| Cough duration (weeks) | 7 (4-8) | 8 (4-8) |
| Dry cough | 28 (43.1%) | 8 (40%) |
| **Cough severity evaluation** |  |  |
| Cough VAS | 60.0 (42.5-80.0) | 60.0 (45.0-79.5) |
| Daytime cough scores | 3 (3-4) | 3 (3-3.5) |
| Night-time cough scores | 1 (1-2) | 2 (1-2) |
| LCQ scores | 13.0 (12.0-13.9) | 12.6 (12.1-13.5) |
| **Blood** |  |  |
| Neutrophils (%) | 59.55 (54.03-66.18) | 60.30 (54.10-67.80) |
| Eosinophils (%) | 1.55 (1.03-2.85) | 1.70 (0.90-3.10) |
| Lymphocytes (%) | 31.38 ± 7.82 | 30.54 ± 7.87 |
| **Induced sputum** |  |  |
| Neutrophils (%) | 62.50 (34.19-84.00) | 61.50 (26.00-81.63) |
| Macrophages (%) | 33.50 (13.75-61.63) | 34.00 (15.88-71.15) |
| Eosinophils (%) | 0.25 (0-1.13) | 0 (0-1.25) |
| Lymphocytes (%) | 1.63 (0.69-3.00) | 2.00 (0.88-3.00) |
| **Spirometry** |  |  |
| FVC% of predicted (%) | 102.3 ± 10.6 | 103.2 ± 10.5 |
| FEV1% of predicted (%) | 100.8 ± 10.0 | 103.6 ± 7.9 |
| MMEF% of predicted (%) | 83.4 ± 18.7 | 86.3 ± 19.1 |
| FEF50% of predicted (%) | 89.2 ± 20.8 | 94.4 ± 24.4 |
| FEF75% of predicted (%) | 80.1 ± 26.5 | 81.9 ± 23.8 |
| **Cough sensitivity evaluation** |  |  |
| LgC2 | 0.89 (0.29-1.19) | 0.59 (0.29-1.19) |
| LgC5 | 1.65 (0.89-2.48) | 1.35 (1.19-2.03) |

Data are expressed as mean ± SD, median (IQR) or n (%). All the differences between PISC and R-PISC at baseline are nonsignificant (*P* > 0.05). BMI = body mass index. VAS = visual analogue scale. FVC: forced vital capacity. FEV1: forced expiratory volume in the first second. MMEF: maximal mid-expiratory flow. FEF: forced expiratory flow. C2 = the lowest concentration of capsaicin that caused 2 or more coughs. C5 = the lowest concentration of capsaicin that caused 5 or more coughs.

**Table S2. IP-10-induced migration of blood lymphocytes *in vitro***

| IP-10 concentration (ng/ml) | HC (n = 34) | PICC (n = 25) | PISC (n = 42) |
| --- | --- | --- | --- |
| 0 | 1 (0) | 1 (0) | 1 (0) |
| 40 | 1.09 (1.01-1.23) | 1.13 (1.04-1.21) | 1.13 (1.04-1.24) |
| 200 | 1.32 (1.08-1.53) | 1.33 (1.18-1.71) | 1.39 (1.24-1.58) |
| 1000 | 1.72 (1.43-1.99) | 2.12 (1.63-2.63)** | 1.83 (1.53-2.16) |

Summarized data, presented as median (IQR), show the relative ratio of IP-10-induced migration of blood lymphocytes to the negative control without IP-10. ***P* < 0.01, compared with the HC group. Otherwise, nonsignificant (*P* > 0.05) between different groups. IP = interferon-γ-inducible protein.

**Table S3. Cytokine concentrations in plasma**

|  | HC (n = 35) | PICC (n = 52) | PISC (n = 56) | R-PISC (n = 17) |
| --- | --- | --- | --- | --- |
| IFN-γ concentration in plasma (pg/ml) | 10.24 (9.13-12.55) | 10.24 (9.25-11.88) | 10.80 (9.25-12.55)* | 8.20 (6.98-9.29)### |
| IL-6 concentration in plasma (pg/ml) | 1.21 (1.21-1.52) | 1.21 (1.04-1.47) | 1.43 (1.11-1.85) | 1.02 (1.02-1.47)### |
| IFN-α concentration in plasma (pg/ml) | 2.10 (1.81-2.41) | 2.10 (1.70-2.48) | 2.23 (1.70-3.06) | 2.18 (1.87-2.50) |
| IL-1β concentration in plasma (pg/ml) | 3.39 (2.70-3.39) | 3.39 (3.25-3.85) | 3.85 (3.39-4.17)** | 3.25 (3.25-3.25)### |

Data are expressed as median (IQR). **P* < 0.05; ***P* < 0.01; compared with the HC group. ###*P* < 0.001; compared with the PIC group. Otherwise, nonsignificant (*P* > 0.05) between different groups. IL = interleukin. IFN = interferon.

**Table S4. Correlations of blood T lymphocyte sub-populations with LgC5**

|  | LgC5 |
| --- | --- |
| The proportion of IFN-γ^+^ T lymphocytes in blood T lymphocytes | -0.262** |
| The proportion of CD8^+^IFN-γ^+^ T lymphocytes in blood T lymphocytes | -0.275** |
| The proportion of CXCR3^+^IFN-γ^+^ T lymphocytes in blood T lymphocytes | -0.194* |
| The proportion of HLADR^+^IFN-γ^+^ T lymphocytes in blood T lymphocytes | -0.194* |

Data are presented as Spearman's r values. **P* < 0.05, ***P* < 0.01. n = 160. C5 = the lowest concentration of capsaicin that caused 5 or more coughs. IL = interleukin. IFN = interferon. CCR4 = CC chemokine receptor 4. CD = cluster of differentiation. CXCR3 = CXC chemokine receptor 3. HLADR = human leukocyte antigen DR.

**Table S5. Compounds and materials used in this study**

| Compounds and materials | Reference | Source |
| --- | --- | --- |
| Phosphate buffered saline (PBS) | PTG0021 | Boster Biotechnology |
| FcR Blocking Reagent, human | 130-059-901 | Miltenyi Biotechnology |
| PE Mouse Anti-Human IFN-γ | 559327 | BD Bioscience |
| PE Mouse IgG1, κ Isotype Control | 551436 | BD Bioscience |
| PerCP-Cy™5·5 Mouse Anti-Human CD194/CCR4 | 560726 | BD Bioscience |
| PerCP-Cy™5·5 Mouse IgG1, κ Isotype Control | 400149 | BD Bioscience |
| BV510 Mouse Anti-Human HLA-DR | 563083 | BD Bioscience |
| BV510 Mouse IgG2a, κ Isotype Control | 563483 | BD Bioscience |
| Alexa Fluor® 488 Mouse Anti-Human CD183/CXCR3 | 558047 | BD Bioscience |
| Alexa Fluor® 488 Mouse IgG1, κ Isotype Control | 557721 | BD Bioscience |
| PE-Cy7 Mouse Anti-Human CD3 | 563423 | BD Bioscience |
| PE-Cy7 Mouse IgG1, κ Isotype Control | 25-4714-42 | Thermo Fisher |
| BV421 Mouse Anti-Human CD4 | 562424 | BD Bioscience |
| BV421 Mouse IgG1, κ Isotype Control | 562438 | BD Bioscience |
| APC-Cy7 Mouse Anti-Human CD8 | 557834 | BD Bioscience |
| APC-Cy7 Mouse IgG1, κ Isotype Control | 557873 | BD Bioscience |
| Fixable Viability Stain 780 (FVS780) | 565388 | BD Biosciences |
| Erythrocytes Lysing Buffer | 349202 | BD Biosciences |
| Cytofix/Cytoperm solution | 554714 | BD Biosciences |
| The total protein assay kit | A045-3 | Nanjing Jiancheng Bioengineering Institute |
| The uric acid assay kit | A22181 | Thermo Fisher |
| Lactate dehydrogenase assay kit | A020-2-2 | BD Bioscience |
| Leukocyte Activation Cocktail, with BD GolgiPlug™ | 550583 | BD Bioscience |
| Recombinant Human IP-10 (CXCL10) | 300-12 | Peprotech |
| Fetal bovine serum | 10099141 | Gibco |
| Sodium Azide | S2002 | Sigma-aldrich |
| Dithiothreitol | D9163-5g | Sigma-aldrich |
| Hematoxylin-Eosin | BA-4098 | BASO Biotechnology |
| Trypan Blue Solution, 0·4% | 15250061 | Gibco |
| Transwell chambers | 3421 | Corning Costar |
| Ficoll-Paque™ PLUS Media | 17-1440-03 | GE hyclone |
| Calcium-free and magnesium-free Hank’s Balanced Salt Solution | C0219 | Jinuo Biotechnology |
| RPMI 1640 Medium | 72400054 | Gibco |
| Bovine serum albumin | B2064-100G | Sigma-aldrich |
| Capsaicin | 404-86-4 | Sigma-aldrich |
| Human cytokine magnetic bead panel | LXSAHM-12 | R&D Systems |
